# Reducing the number of parameters in 1D arterial blood flow modeling: less is more for patient-specific simulations

**DOI:** 10.1152/ajpheart.00857.2014

**Published:** 2015-04-17

**Authors:** Sally Epstein, Marie Willemet, Phil J. Chowienczyk, Jordi Alastruey

**Affiliations:** ^1^Division of Imaging Sciences and Biomedical Engineering, St. Thomas' Hospital, King's College London, London, United Kingdom; and; ^2^Department of Clinical Pharmacology, St. Thomas' Hospital, King's College London, London, United Kingdom

**Keywords:** 1D modeling, aortic pulse wave, digital pulse wave, windkessel model, hypertension

## Abstract

Patient-specific one-dimensional (1D) blood flow modeling requires estimating model parameters from available clinical data, ideally acquired noninvasively. The larger the number of arterial segments in a distributed 1D model, the greater the number of input parameters that need to be estimated. We investigated the effect of a reduction in the number of arterial segments in a given distributed 1D model on the shape of the simulated pressure and flow waveforms. This is achieved by systematically lumping peripheral 1D model branches into windkessel models that preserve the net resistance and total compliance of the original model. We applied our methodology to a model of the 55 larger systemic arteries in the human and to an extended 67-artery model that contains the digital arteries that perfuse the fingers. Results show good agreement in the shape of the aortic and digital waveforms between the original 55-artery (67-artery) and reduced 21-artery (37-artery) models. Reducing the number of segments also enables us to investigate the effect of arterial network topology (and hence reflection sites) on the shape of waveforms. Results show that wave reflections in the thoracic aorta and renal arteries play an important role in shaping the aortic pressure and flow waves and in generating the second peak of the digital pressure and flow waves. Our novel methodology is important to simplify the computational domain while maintaining the precision of the numerical predictions and to assess the effect of wave reflections.

persistently elevated blood pressure (hypertension) is now identified as the most important single cause of mortality worldwide. By 2025 it is predicted that 1.56 billion people will have hypertension (22). The correlation between hypertension and independent biomarkers has been extensively analyzed (25, 47). However, understanding how properties of the cardiovascular system affect the aortic blood pressure waveform and its relation to the pressure waveform in the upper limb (where blood pressure is routinely measured) has not yet been fully understood (20). One-dimensional (1D) arterial blood flow modeling has the potential to improve this understanding, specially if patient-specific simulations could be achieved.

Several studies have shown the ability of the nonlinear 1D equations of blood flow in compliant vessels to capture the main features of pressure and flow waves in the aorta and other large arteries (26, 30, 32, 38, 50). 1D models offer good accuracy with considerable less computational cost than equivalent three-dimensional (3D) models (15, 52). However, a major drawback of 1D modeling is that the determination of model input parameters from clinical data is a very challenging inverse problem (35, 36). In the 1D formulation, the arterial network is decomposed into arterial segments connected to each other at nodes. The larger the number of arterial segments in a given 1D model, the greater the number of model input parameters that need to be estimated. Thus a method of minimizing the number of 1D model arterial segments should help maximize the percentage of input parameters that can be estimated from clinical measurements and, hence, that can be patient specific.

Within the field of multibranched 1D modeling, there has yet to be a consensus reached in terms of the optimum number of arterial segments necessary to obtain good quality pressure and flow measurements at the aorta. The number of arterial segments used in 1D modeling has increased in recent years from 55 to over 4 million (7, 11, 30, 33, 38, 42). Obviously, with an increasing number of arterial segments the more challenging it becomes to estimate a greater percentage of the total number of input parameters from available patient-specific data. Running a distributed 1D model requires geometric and material properties for each arterial segment, a blood inflow waveform, and outflow boundary conditions for every single terminal vessel. Thus with each generation of bifurcations in the arterial tree the number of input parameters that need to be prescribed increases exponentially.

Lumped parameter models of the cardiovascular system are also commonly used to simulate arterial blood flow. They were introduced by Otto Frank in 1899 (17) under the guise of a “hydraulic windkessel,” which consists of a resistor and capacitor in series. The windkessel model is adept at portraying the exponential decay in diastole, but it is less able to describe the pressure waveform during the systolic phase of the cardiac cycle (39). More elaborate combinations of resistors, capacitors, and impedances have been suggested to improve fitting in systole (24, 31, 43). However, with the drop in the space dimension, the windkessel model neither accounts for wave propagation and reflection nor can simulate the effect of pulse wave velocity (PWV), which is assumed to be infinite. Wave reflections play an important role in shaping the pressure waveform and central PWV has been identified as a highly valuable marker of arterial stiffness, which is being used in the classification and diagnosis of hypertension (27). Wave propagation and reflection can be studied using 1D modeling (51).

The aim of this study is to provide a new methodology for investigating the minimum number of tapered arterial segments required to simulate, using nonlinear 1D modeling, the blood pressure and flow waveforms in the aorta and digital artery in the hand, where the upper limb pressure waveform is usually measured noninvasively. This is achieved by lumping peripheral 1D model branches into windkessel models that preserve the net resistance and total compliance of the original model. In addition, this methodology enables the analysis of the effect of network topology and, hence, reflections sites on the shape of pressure and flow waves. Our novel methodology is tested for the aorta of a 55-artery model under both normotensive and hypertensive conditions and for the digital artery of a 67-artery model under normotensive conditions (Fig. 1).

**Fig. 1.**
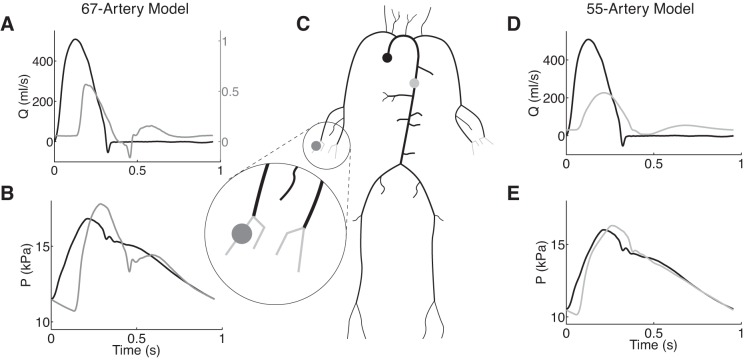
*A*: flow waveform prescribed at the aortic root as a reflective boundary condition (black), and simulated flow waveform at the digital artery (gray) for the 67-artery model. *B*: simulated pressure waveforms at the aortic root (black) and digital artery (gray) for the 67-artery model. *C*: schematic representation of the 55-artery 1-dimensional (1D) model (black). The circled region shows additional anatomical model of the hand included in the 67-artery model (grey), containing vessels of the superficial palmar arch and digital arteries. *D*: flow waveform prescribed at the aortic root as a reflective boundary condition (black), and simulated flow waveform at the thoracic aorta (grey) for the 55-artery model. *E*: simulated pressure waveforms for the 55-artery model at the aortic root (black) and thoracic aorta (gray).

## METHODS

We begin by describing the 1D formulation (see *1D Formulation*) and the 55 and 67-artery models considered in this study (see *55-Artery Model* and *67-Artery Model*). We then introduce our novel technique for lumping peripheral 1D model branches into windkessel 0D models (see *Reducing the Number of Arterial Segments*). Lastly, we describe the error metrics used to analyze our results (see *Error Metrics*).

### One-Dimensional Formulation

In the 1D formulation, each segment is modeled as a deformable cylindrical tube whose properties can be described by a single axial coordinate *x*. Assuming the tube to be impermeable and the fluid to be incompressible and Newtonian, conservation of mass and momentum applied to a control volume of the tube yields (16, 40)

(1A) $$\frac{\partial A}{\partial t}+\frac{\partial \left(AU\right)}{\partial x}=0,$$

(1B) $$\frac{\partial U}{\partial t}+U\frac{\partial U}{\partial x}=-\frac{1}{\rho }\frac{\partial P}{\partial x}+\frac{f}{\rho A},$$

where *t* is the time; ρ = 1,050 kg/m^3^ is the density of the blood; *A*(*x*, *t*) is the luminal cross-sectional area, assumed to be circular; and *U*(*x*, *t*) and *P*(*x*, *t*) are the axial blood flow velocity and pressure, respectively, averaged over *A*. The frictional force per unit length is given by *f*(*x*, *t*) = −2(ξ + 2)πμ*U*, with ξ the polynomial order of the velocity profile and μ = 4 mPa·s the viscosity of the blood. We assume a close to plug flow with ξ = 9 (41).

To close the system of nonlinear governing *Eqs. 1A* and *1B* we need a pressure-area relationship, known as tube law. In this model we assume the arterial wall to be a linear elastic, thin, incompressible and homogenous solid, which yields the tube law (34)

(2) $$P=P_{\text{d}}+\frac{\beta }{A_{\text{d}}}\left(\sqrt{A}-\sqrt{A_{\text{d}}}\right),$$

where *A*_d_ is the cross-sectional area at diastolic pressure (*P*_d_). The parameter β characterizes the material properties of the vessel through

(3) $$\beta =\frac{4}{3}\sqrt{\pi }Eh,$$

with *E* the elastic modulus and *h* the thickness of the vessel wall. β Is related to the PWV, denoted by *c*, through

(4) $$c=\sqrt{\frac{\beta }{2\rho A_{\text{d}}}}A^{1/4}.$$

*Equations 1* and *2* are solved numerically using a discontinuous Galerkin scheme with a spectral/*hp* spatial discretization (4).

### 55-Artery Model

**Fig. 2.**
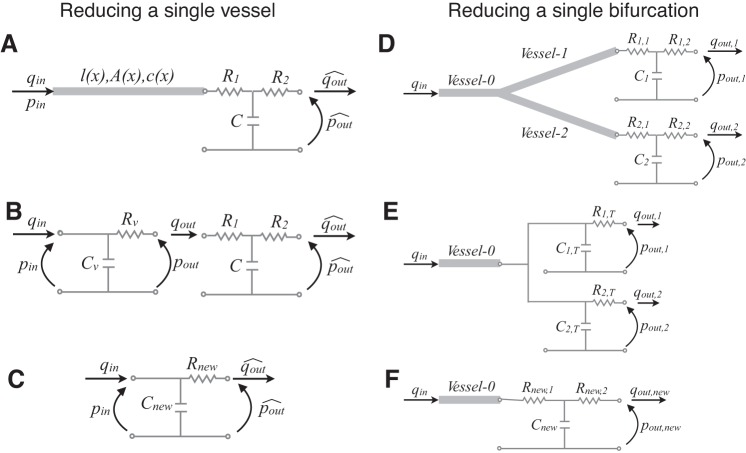
*A–C*: reduction of a nonlinear 1D model single vessel attached to a 3-element windkessel model (*R*_1_–*C–R*_2_; *A*) into a single 2-element windkessel model (*C*_new_–*R*_new_; *C*), via an intermediary stage (*B*) in which the 1D vessel is simplified into a 2-element windkessel model (*C*_v_–*R*_v_). The 1D vessel is characterized by a length *l*, a cross-sectional area *A*(*x*) and a pulse wave velocity *c*(*x*). *D–F*: reduction of a nonlinear 1D model single bifurcation coupled to 3-element windkessel models (*R*_1_–*C–R*_2_; *D*) into a nonlinear 1D model single vessel coupled to a 3-element windkessel model (*R*_new,1_–*C*_new_–*R*_new,2_; *F*). In the intermediary stage (*E*), the daughter vessels 1 and 2 and their outlet windkessel models are transformed into two 2-element windkessel models (*C*_T_–*R*_T_). *q*_in_(*t*): inflow; *q*_out_(*t*): outflow; *p*_in_(*t*): inflow pressure; *p*_out_: outflow pressure.

The baseline 1D arterial network considered in this work consists of the larger 55 arteries (Fig. 1*C*, black) with the properties of a normotensive human. Each arterial segment is described by a set of three independent parameters (length, luminal cross-sectional area at diastolic pressure, and diastolic PWV), as described in detail in Ref. 4. Energy losses are neglected at bifurcations and the initial conditions are (*A,U*, *P*) = [*A*_0_(*x*), 0, 0] in all segments, with *A*_0_ the luminal area that yields *A*_d_ at diastolic pressure, *P*_d_. The flow at the aortic root (Fig. 1*D*) is prescribed as a reflective boundary condition. The outlet of each terminal vessel is coupled to a three-element windkessel model, with two peripheral resistances (*R*_1_ and *R*_2_), a compliance (*C*) and an outflow pressure pout^ (Fig. 2*A*). All four parameters are assumed to be constant, but with different values in each terminal vessel. The resistance *R*_1_ is equal to the characteristic impedance *Z*_0_ at the end point of the vessel, with

(5) $$Z_{0}=\frac{\rho c}{A_{\text{d}}}.$$

Figure 1, *D* and *E*, shows the flow and pressure waveforms at the aortic root and thoracic aorta of the baseline model. We also considered a hypertensive model obtained by increasing arterial stiffness in all vessels of the baseline model and peripheral resistances in all windkessel models. Both PWV (which is related to the stiffness parameter β by *Eq. 4*) and the total peripheral resistance in each terminal windkessel (*R*_1_ + *R*_2_) are increased by a factor of 1.5.

### 67-Artery Model

We extended the baseline model by including the larger arteries of the hand using previously published data (Table A1 in Ref. 1) (see Fig. 1*C*, gray). Each hand consists of the superficial palmar arch and four digital arteries attached to the ulnar and radial arteries of the baseline model. Figure 1, *A* and *B*, shows the flow and pressure waveforms at the aortic root and digital artery of the 67-artery model. Pressure and flow in all four digital arteries are almost identical.

### Reducing the Number of Arterial Segments

The baseline model consists of the aorta and five generations of arterial bifurcations (Fig. 1*C*, black). We reduced the 55 arterial segments of this model using two procedures. In the first one, each successive reduction in the number of segments was achieved by decreasing the number of generations of bifurcations. For example, by eliminating the fifth generation of bifurcations, the arterial network is reduced to 53 arteries, since the right interosseous and right lower ulnar arteries (the only vessels at the 5th generation of bifurcations) are lumped into a single three-element windkessel model coupled to the end point of the right upper ulnar.

In the second procedure, the number of arteries of the model containing the aorta up to the first generation of bifurcations was reduced by successively lumping each pair of the most peripheral vessels into a three-element windkessel model. For example, the model up to the first generation of bifurcations (with 21 vessels) was reduced to 19 vessels by combining the left and right common iliac arteries into a three-element windkessel model coupled to the lower abdominal aorta.

The 67-artery model (Fig. 1*C*, grey) was reduced to a model consisting of only the arterial segments of the upper right limb and ascending aorta, which is similar to the model of Karamanoglu et al. (21). Similarly to the 55-artery model, we reduced the number of arteries by first successively decreasing the number of generations of bifurcations and then systematically reducing the number of aortic segments. Thus the 67-artery model was reduced up to the 19-artery model shown in Fig. 6*F*. This reduction enables the evaluation of the waveform at the digital artery (a peripheral measuring site) throughout the systematic reduction of arteries.

#### Reducing a 1D model terminal branch into an equivalent 0D model.

We can lump a 1D model vessel coupled to a three-element windkessel model (with resistances *R*_1_, *R*_2_ and compliance *C*, Fig. 2*A*) into an equivalent two-element windkessel model (Fig. 2*C*) with resistance (*R*_new_) and compliance (*C*_new_). These are calculated as

(6A) $$R_{\text{new}}=R_{\text{2}}+R_{\text{1}}+R_{\text{v}},$$

(6B) $$C_{\text{new}}=\frac{C_{\text{v}}R_{\text{2}}+C_{\text{v}}R_{\text{1}}+CR_{\text{2}}+R_{\text{v}}C_{\text{v}}}{R_{\text{2}}+R_{\text{1}}+R_{\text{v}}},$$

where *R*_v_ and *C*_v_ are, respectively, the resistance and compliance of the 1D model vessel (Fig. 2*B*) given by

(7) $$\begin{array}{rr} R_{\text{v}}=2\left(\xi +2\right)\pi \mu K_{3}, & K_{3}=\int_{0}^{l}\frac{1}{A_{\text{d}}^{2}}dx,\\ C_{\text{v}}=\frac{K_{1}}{\rho }, & K_{1}=\int_{0}^{l}\frac{A_{\text{d}}}{c_{\text{d}}^{2}}dx. \end{array}$$

*Equations 6A*, *6B*, and *7* can be derived by neglecting nonlinearities and inertia terms and assuming that pulse wave transit times within a vessel are much smaller than the duration of the cardiac cycle. Further details are given in the appendix.

#### Reducing a 1D model single bifurcation into an equivalent 0D model.

We consider an arterial bifurcation with the parent vessel denoted by the index 0 and the daughter vessels by the indexes 1 and 2 (Fig. 2*D*). Firstly, we apply the methodology described in *Reducing a 1D model terminal branch into an equivalent 0D model* to transform each daughter vessel into two parallel RC-windkessel models, with elements *R*_1_,_T_, *C*_1,T_, and *R*_2,T_, *C*_2,T_ for daughter vessels 1 and 2, respectively (Fig. 2*E*). We can then combine these two 0D models into a single three-element windkessel model, with resistances *R*_new,1_ and *R*_new,2_ and compliance *C*_new_ (Fig. 2*F*). The total peripheral resistance of the new 0D model is calculated as

(8) $$R_{\text{new},1}+R_{\text{new},2}=\frac{1}{\frac{1}{R_{1,\text{T}}}+\frac{1}{R_{2,\text{T}}}},$$

where *R*_new,1_ is set to be equal to *Z*_0_ (*Eq. 5*) of the parent vessel to minimize wave reflections (2). The total peripheral compliance of the new three-element windkessel is calculated as

(9) $$C_{\text{new}}=C_{1,\text{T}}+C_{2,\text{T}}.$$

### Error Metrics

The pressure and flow waveforms generated by the reduced models were compared with those generated by the complete 55- or 67-models using the following relative error metrics:

(10) $$\begin{array}{rr} \in_{P,\text{avg}}=\frac{1}{N_{t}}\overset{N_{t}}{\underset{i=1}{\sum }}\left|\frac{P_{i}^{\text{R}}-P_{i}^{\text{C}}}{P_{i}^{\text{C}}}\right|, & \in_{Q,\text{avg}}=\frac{1}{N_{t}}\overset{N_{t}}{\underset{i=1}{\sum }}\left|\frac{Q_{i}^{\text{R}}-Q_{i}^{\text{C}}}{\max_{j}\left(Q_{j}^{\text{C}}\right)}\right|,\\ \in_{P,\text{sys}}=\frac{\max_{i}\left(P_{i}^{\text{R}}\right)-\max_{i}\left(P_{i}^{\text{C}}\right)}{\max_{i}\left(P_{i}^{\text{C}}\right)}, & \in_{Q,\text{sys}}=\frac{\max_{i}\left(Q_{i}^{\text{R}}\right)-\max_{i}\left(Q_{i}^{\text{C}}\right)}{\max_{i}\left(Q_{i}^{\text{C}}\right)},\\ \in_{P,\text{dias}}=\frac{\min_{i}\left(P_{i}^{\text{R}}\right)-\min_{i}\left(P_{i}^{\text{C}}\right)}{\min_{i}\left(P_{i}^{\text{C}}\right)}, & \in_{Q,\text{dias}}=\frac{\min_{i}\left(Q_{i}^{\text{R}}\right)-\min_{i}\left(Q_{i}^{\text{C}}\right)}{\max_{i}\left(Q_{i}^{\text{C}}\right)}, \end{array}$$

with *N*_*t*_ the number of time points where the comparison is made. For each time point *i*, *P*_*i*_^*C*^ and *P*_*i*_^*R*^ are the pressures at the same spatial location of the complete (*C*) and reduced (*R*) model, respectively. The corresponding flows are *Q*_*i*_^*C*^ and *Q*_*i*_^*R*^. The metrics $\in_{P,\text{avg}}\;\text{and}\;\in_{Q,\text{avg}}$ are the average relative errors in pressure and the flow, respectively, over one cardiac cycle; $\in_{P,\text{sys}}\;\text{and}\;\in_{Q,\text{sys}}$ are the errors for systolic pressure and flow; and $\in_{P,\text{dias}}\;\text{and}\;\in_{Q,\text{dias}}$ are the errors for diastolic pressure and flow. To avoid division by small values of the flow, we normalized the flow errors by the maximum value of the flow over the cardiac cycle, max_*j*_(*Q*_*j*_^*C*^).

## RESULTS

We applied our method for reducing the number of 1D model arterial segments described in *Reducing the Number of Arterial Segments* to the normotensive (see *Normotensive 55-Artery Model*) and hypertensive (see *Hypertensive 55-Artery Model*) 55-artery models, as well as the normotensive 67-artery model (see *Normotensive 67-Artery Model*). In all cases we analyzed the effect of decreasing the number of arterial segments on the pressure at the aortic root. For the 55-artery model we also investigated the pressure and flow at the midpoint of the thoracic aorta. For the 67-artery model we also studied the pressure and flow waveforms at the digital artery in the hand. The aortic-root flow is not studied for any model, since it is enforced as the inflow boundary condition.

### Normotensive 55-Artery Model

**Fig. 3.**
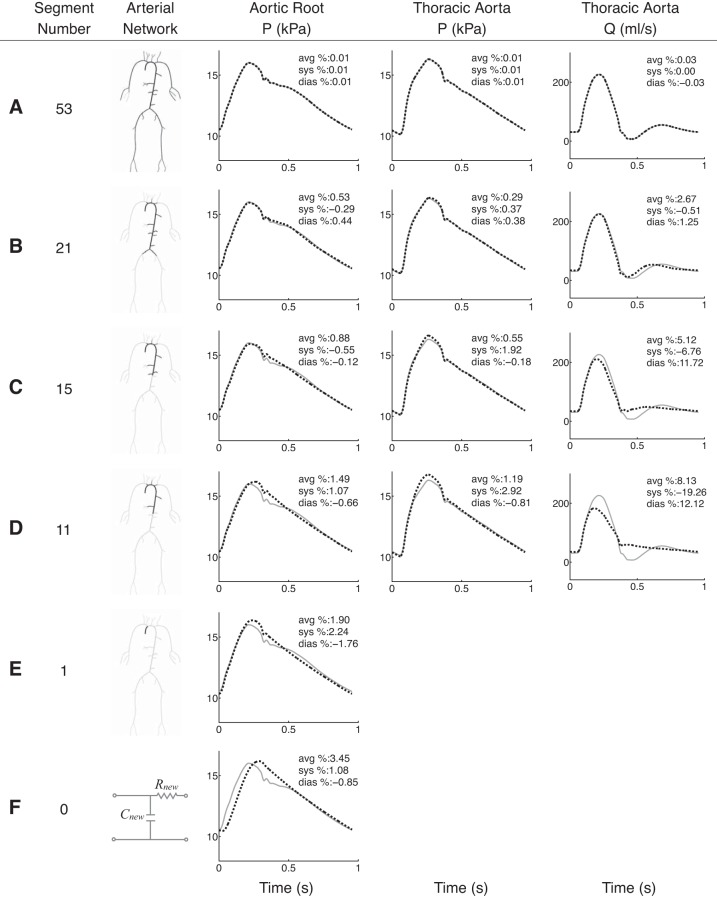
*A–F*: pressure (*P*) and flow (*Q*) waveforms at the aortic root and midpoint of the thoracic aorta of the normotensive 55-artery model (solid grey) and several reduced models (dashed black), 1 for each row. The number of segments and the arterial topology for each reduced model are given in the 1st 2 columns. The aortic-root pressure waveform calculated by a 2-element windkessel model of the whole systemic circulation is shown in *F*. Average (avg), systolic (sys) and diastolic (dias) relative errors are indicated in the *top right corner* of each plot.

Figure 3 compares the aortic waveforms simulated by six reduced models (dashed black) with those produced by the baseline model (solid grey) under normotensive conditions. Average, systolic and diastolic errors are provided in each plot relative to the baseline model. For each reduced model in Fig. 3, *A–E*, the number of segments is shown in the first column and the model topology is illustrated in the second. Diastolic, systolic, and average relative errors in aortic pressure are <3% for any reduced model, even for the single-vessel model shown in Fig. 3*E*. Relative errors in the flow waveform at the thoracic aorta are greater than in pressure but remain smaller than 20% (9% for the average relative error).

Considerable discrepancies between reduced and baseline models appear if 15 or less segments are included. These models, which do not include the superior mesenteric and renal arteries, lead to the following features of the aortic waveform not being captured as well as in the baseline model (Fig. 3, *C* and *D*): the magnitude of the dicrotic notch and the time of peak systole at the root, and the magnitude of systolic pressure, the time and magnitude of the peak flow, and the oscillatory flow in diastole at the thoracic aorta. However, all reduced models capture well the time and magnitude of the feet of baseline pressure and flow waves. Moreover, they produce pressure and flow waveforms in the second half of diastole that are similar to those simulated by the complete 55-artery model.

Figure 3*F* shows the aortic-root pressure waveform calculated by a two-element windkessel model with a resistance and compliance equal to, respectively, the net peripheral resistance and total compliance of the baseline model. This 0D model is able to capture precisely the decay in aortic pressure in diastole but is unable to describe well pressure in systole.

**Fig. 4.**
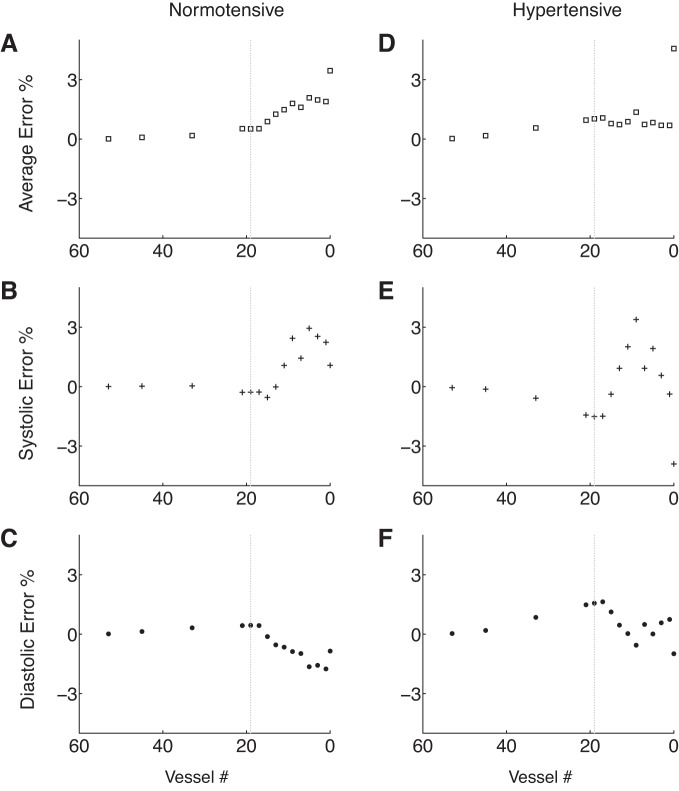
Evolution of the average (*A* and *D*), systolic (*B* and *E*), and diastolic (*C* and *F*) relative errors in the pressure waveform at the aortic root of the 55-artery model with the number of arterial segments, under normotensive (*A–C*) and hypertensive (*D–F*) conditions. For each plot, the vertical dashed line corresponds to the 19-artery model: arterial networks on its left (≥19 vessels) include a full aorta, whereas those on its right (<19 vessels) only include portions of it.

Figure 4, *A–C*, shows systolic, diastolic and average relative errors in the pressure at the aortic root with the number of arterial segments. Errors remain <1% for models containing 53 to 21 segments; the latter consists of the aorta and first generation of bifurcations only (see its topology in Fig. 3*B*). Further reductions in the number of segments yield a greater increase in the rate at which errors raise. Qualitatively similar progressions in relative errors are obtained for the flow and pressure at the thoracic aorta.

### Hypertensive 55-Artery Model

**Fig. 5.**
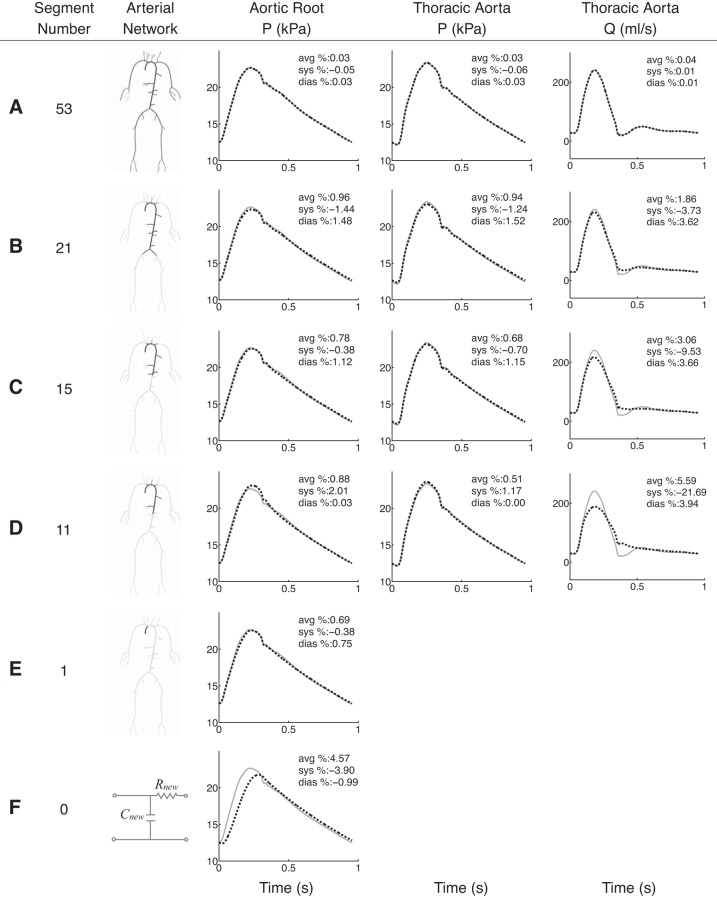
*A–F*: pressure (*P*) and flow (*Q*) waveforms at the aortic root and midpoint of the thoracic aorta of the hypertensive 55-artery model (solid grey) and several reduced models (dashed black), 1 for each row. The number of segments and the arterial topology for each reduced model are given in the 1st 2 columns. The aortic-root pressure waveform calculated by a 2-element windkessel model of the whole systemic circulation is shown in *F*. Average (avg), systolic (sys), and diastolic (dias) relative errors are indicated in the *top right corner* of each plot.

Figure 5 compares the aortic pressure and flow waveforms produced by six reduced models (dashed black) with those simulated by the complete 55-artery model (solid grey) under hypertensive conditions. The topology of the reduced models and the format of the figure are the same as for the normotensive study above. Pulse and mean pressures are greater under hypertensive conditions: the pulse pressure is approximately doubled whereas the mean pressure increases by ∼40%. The amplitude and mean value of the flow change little with simulated hypertension.

Average relative errors in both pressure and the flow remain <2% for models containing 53 to 21 segments, in agreement with the normotensive results (Fig. 4, *A* and *B*). However, eliminating segments of the aorta and first generation of bifurcations from the 21-artery model produces smaller errors under hypertensive conditions. Hypertensive reduced models are able to capture well all features of the aortic pressure waveform that normotensive reduced models were unable to reproduce (compare Figs. 3, *A–E*, and 5, *A–E*). Discrepancies in aortic flow waveforms are similar under both normotensive and hypertensive conditions (compare last column in Figs. 3, *A–D*, and 5, *A–D*). Moreover, similar evolutions of systolic and diastolic errors are observed under both normotensive and hypertensive conditions (Fig. 4).

In agreement with the normotensive results, the aortic-root pressure waveform calculated by the two-element windkessel model of the whole systemic circulation is able to describe well the decay in pressure during diastole but is unable to produce most of the features of the pressure wave in systole (Fig. 5*F*).

### Normotensive 67-Artery Model

**Fig. 6.**
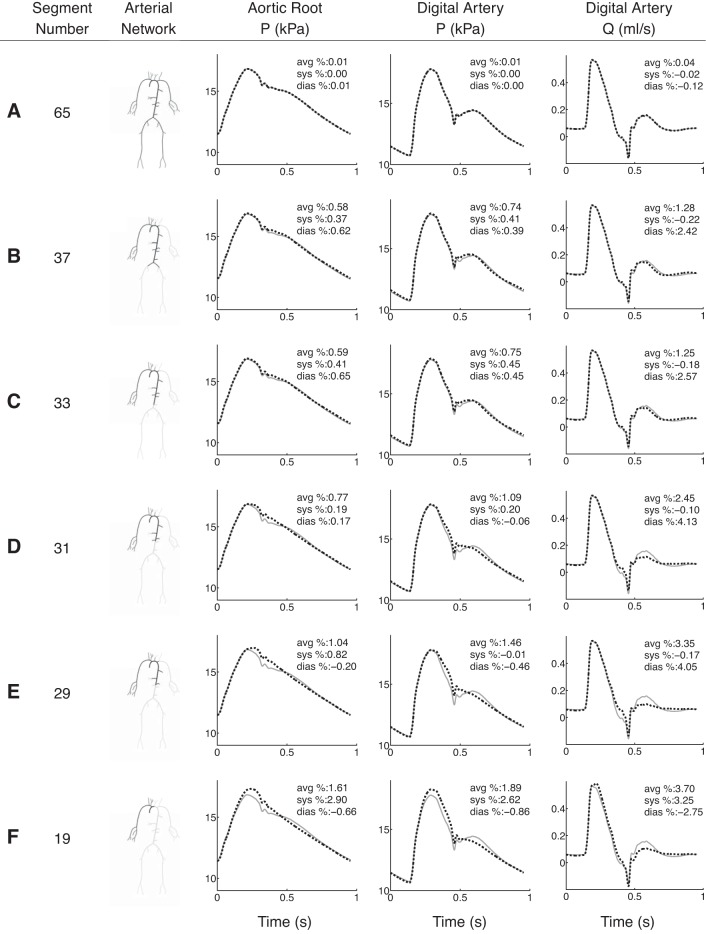
*A–F*: pressure (*P*) and flow (*Q*) waveforms at the aortic root and midpoint of the digital artery of the 67-artery model (solid grey) and several reduced models (dashed black), one for each row. The number of segments and the arterial topology for each reduced model are given in the 1st 2 columns. Average (avg), systolic (sys), and diastolic (dias) relative errors are indicated in the *top right corner* of each plot.

Figure 6 compares the aortic pressure and flow waveforms produced by six reduced models (dashed black) with those simulated by the complete 67-artery model (solid grey). Pulse and mean pressures differ from those of the baseline 55-artery model by −9 and 13% at the aortic root (Fig. 1). This is due to the total resistance and compliance being altered by the extended arterial network in the hands. With segment reductions, average systolic and diastolic relative errors of central aortic and digital arterial pressure remain <3% for models containing 65 to 19 segments. Relative errors in the flow at the digital artery remain <5%.

**Fig. 7.**
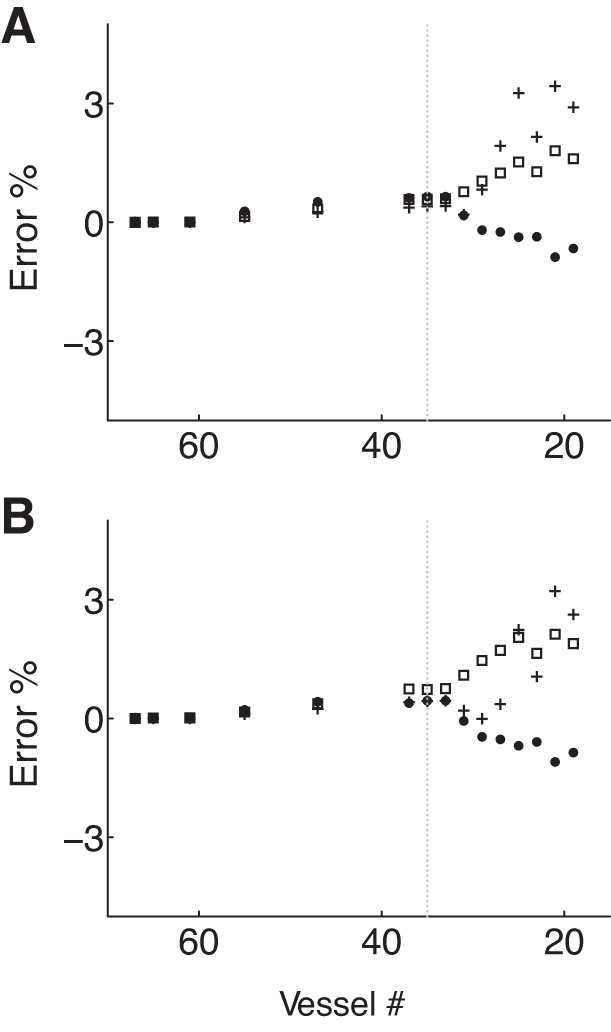
Evolution of the average (□), systolic (+), and diastolic (●) relative errors in the pressure waveform at the aortic root (*A*) and digital artery (*B*) with the number of arterial segments in the 67-artery model. Vertical dashed lines correspond to the 35-artery model: arterial networks displayed on the *left* and on the line include a full aorta while those on the *right* only include portions of it.

Reduced 67-artery models with <33 vessels do not capture the following features at the central aorta: magnitude of the dicrotic notch and the time and magnitude of peak systole (Fig. 6, *D–F*). At the digital artery these reduced models no longer capture the time of arrival of the second pressure peak, the magnitude of the first pressure peak, and the oscillatory flow in diastole. Figure 7 shows average, systolic, and diastolic relative errors in the pressure at the aortic root (*A*) and digital artery (*B*) with the number of arterial segments. The largest divergence between the complete and reduced models is seen if less than 33 vessels are included; i.e., if aortic segments are lumped into windkessel models.

## DISCUSSION

In this study we have presented a novel methodology for investigating the minimum number of arterial segments required to simulate precisely blood pressure and flow waveforms at multiple arterial sites using nonlinear 1D modeling. By systematically lumping terminal 1D model segments into 0D windkessel models that preserve the net resistance and total compliance of the original model (Fig. 2), we can study the effect on central and peripheral waveforms of reducing the number of arterial segments and, hence, input parameters. We have applied our methodology to investigate the effect of network topology (and hence reflection sites) on the shape of pressure and flow waves in the aorta and digital artery in the hand. To carry out this study we have used a nonlinear 1D model of blood flow in the 55 larger systemic arteries of the human, under normotensive and hypertensive conditions, and an extended 67-artery model that includes the larger arteries in the hand, under normotensive conditions (Fig. 1).

We have focused on analyzing *1*) aortic hemodynamics, because central (aortic) pressure is an important determinant of cardiovascular function; and *2*) peripheral hemodynamics in the digital arteries, because the pressure and volume pulses at the digital artery can be measured continuously and noninvasively in the clinic; e.g., using the Finapres device (18) and photoplethysmography (5, 8), respectively. Our simulated aortic and digital pressure and flow waveforms contain the main features observed in vivo (Fig. 1).

### Optimizing Arterial Network Topology

The 55-artery model consists of the aorta and other large arteries up to the fifth generation of bifurcations (Fig. 1) and is determined by 227 independent parameters. The 67-artery model consists of the aorta and other large arteries up to the sixth generation of bifurcations (which include vessels of the palmar arch and digital arteries Fig. 1) and is defined by 271 independent parameters. Each arterial segment is described by three independent parameters (length, luminal cross-sectional area at diastolic pressure, and diastolic PWV), while two additional parameters are required to model each peripheral arterial segment (peripheral resistance and compliance). The remaining independent parameters are the density and viscosity of blood, the shape of the velocity profile, the diastolic pressure, the inflow boundary condition and the outflow pressure at terminal windkessel models.

By subsequently trimming the 55-artery model of generations of bifurcations, the number of arterial segments is reduced to 21, if only arterial vessels up to the first generation of bifurcations are simulated using 1D modeling. As a result, the 227 input parameters of the initial model are reduced to 91. Such a considerable reduction in the number of parameters yields aortic pressure and flow waveforms that contain all the features of the 55-artery model with errors <4% relative to the initial model (Figs. 3*B* and 5*B*). Reducing the 67-artery model (271 input parameters) to the first generation of bifurcations (37 arteries, 171 parameters), with all segments of the right upper limb preserved, results in relative errors <1% in pressure at the central and peripheral measuring sites and <3% in the flow at the peripheral measuring site, with all errors relative to the initial model (Fig. 6*B*). These results suggest that our original 55-artery (67-artery) models are overparameterized for simulating aortic (and digital) waveforms.

The focus of previous studies using 1D modeling (7, 11, 30, 33, 38, 42) was not confined to the simulation of aortic and upper-limb pulse waveforms. However, according to our study, 1D modeling of these pulse waveforms may not require the large number of arterial segments considered in those studies. Instead, our results favor the use of reduced models that consider only the arteries of interest, e.g., the model of Karamanoglu et al. (21) for the ascending aorta and upper-limb vessels and the model of Willemet et al. (50) for the lower-limb vessels. This result has important implications for patient-specific 1D modeling. A reduction in the number of 1D model arterial segments decreases the number of input parameters that need to be prescribed, which should facilitate the estimation of a greater percentage of the total number of parameters from clinical measurements. Lowering the number of parameters, therefore, should increase the degree of patient-specific modeling that can be achieved to simulate central and upper-limb hemodynamics. However, it is important to note that the degree of applicability of 1D models for patient-specific modeling remains at a pilot stage. As such, the task of clinical estimation of 1D model parameters may still be challenging, even if using the suggested lumping approach.

Our results also contribute towards understanding the role of pulse wave reflections in shaping the aortic (see *Effect of Wave Reflections on Aortic Pressure and Flow Waveforms*) and digital (see *Effect of Wave Reflections on Digital Pressure and Flow Waveforms*) pressure and flow waves.

### Effect of Wave Reflections on Aortic Pressure and Flow Waveforms

The excellent agreement between the 55-artery model and the reduced 21-artery model containing segments up to the first generation of bifurcations (Figs. 3*B* and 5*B*) suggests that the effect of multiple reflection sites downstream vessels of the first generation of bifurcations can be lumped into single reflection sites at the end of these vessels. Further reduction in the number of arterial segments by lumping aortic segments considerably increases relative errors once the superior mesenteric and renal arteries are removed (Fig. 3, *C–E*). These observations are supported by in vivo studies showing that reflected waves generated artificially by total occlusions in the aorta distal to the renal arteries have no discernible influence on the pressure and flow waveforms at the ascending aorta (23, 45). It is also in agreement with the outcome of numerical studies that used different tools of pulse wave analysis (3, 21). Our numerical results are also consistent with the horizon effect hypothesis (13), which states that aortic reflected waves are an amalgam of individual reflections from multiple reflection sites, with the nearer site contribution at full strength and sites further away having their contributions attenuated.

Further reductions in the number of 1D model segments of the 55-artery model by lumping aortic segments and arteries that branch off the aorta into 0D models, starting from the iliac bifurcation, yields relative errors in aortic pressure <5% (Fig. 4). Relative errors in aortic-root pressure remain <5% even if all the 55 segments of the baseline model are integrated into Frank's (17) two-element windkessel model of the entire arterial system (Figs. 3*F* and 5*F*). Removing aortic segments in the 55-artery model leads to discrepancies in the shape of aortic pressure and flow waveforms, in systole and early diastole, but does not in approximately the last half of diastole. This result suggests that the diastolic part of the pressure waveform along the aorta can be described by a lumped parameter model with only four independent parameters (the inflow from the left ventricle, the total compliance of the systemic circulation, the net resistance at the aortic root, and the outflow pressure), which corroborates in vivo (46, 48) and numerical (51, 52) studies showing that aortic pressure becomes space independent with increasing time in diastole.

Lumping aortic segments of the 55-artery normotensive model yields reduced models that are unable to capture the main features of aortic pressure and flow waves in systole (Fig. 3, *C–F*). Under hypertensive conditions, however, models with a reduced number of aortic segments maintain all features of the aortic-root pressure waveform with relative errors <2% (Fig. 5, *C–E*). This result suggests that individual distal reflection sites affect less the shape of the central pressure wave with hypertension. The following mechanism underlies this result. Greater PWVs in the hypertensive model make more valid the assumption of our reduction methodology of pulse wave transit times within each vessel being much smaller than the duration of the cardiac cycle (see *Remark 1* in appendix). Our additional assumption of negligible inertia effects is also satisfied better in the hypertensive model, since increasing PWV (and hence decreasing arterial compliance) decreases the weight of the flow acceleration term in the conservation of momentum Eq. 1*B* relative to the pressure gradient and viscous terms (51).

### Effect of Wave Reflections on Digital Pressure and Flow Waveforms

In the 67-artery model we systematically lumped aortic segments and branches off the aorta into 0D models while preserving all arterial segments of the upper right limb. For any reduced models, we obtained relative errors in pressure and the flow at the aortic root and digital artery <4.5% (Figs. 6, *A–F* and 7). The model with the least number of segments (19 segments) contains only the ascending aorta and upper-right-limb vessels and has a topology similar to that used by Karamanoglu et al. (21).

Results at the aortic root of the reduced 37-artery model (Fig. 6*B*) confirm our conclusion from the previous section that the effect of multiple reflection sites downstream vessels of the first generation of bifurcations can be lumped into single reflection sites at the end of these vessels.

Pressure and flow waveforms at the digital artery have two peaks, as observed in vivo. The second peak is progressively flattened as aortic segments are lumped (Fig. 6, *B–F*). The greater change in shape occurs if the renal arteries are lumped into 0D models (Fig. 6, *C–E*). This result suggests that wave propagation in the thoracic aorta and renal arteries plays an important role in generating the second peak of the digital pressure and flow waves. This observation corroborates the conclusions from Refs. 9, 10 and is also consistent with the hypothesis in Refs. 12, 29: the second peak of the digital volume pulse (which is closely related to the pressure pulse) is due to pressure waves travelling from the left ventricle to the finger and later smaller reflections from internal mismatching, mainly in the lower body.

### Perspectives

We believe that our methodology could offer valuable insight into optimizing the number of model input parameters that are needed to simulate pressure and flow waveforms in a given region of the systemic circulation other than the aorta and digital artery. This will be useful to *1*) identify which part of the arterial tree is responsible for shaping a particular pressure waveform and *2*) lessen the number of parameters that have to be estimated or taken from the literature to simulate that waveform using 1D modeling. Other studies have considered truncated arterial networks, e.g., to study hemodynamics in the leg or arm, and have shown that arterial pressure and flow waveforms can be efficiently modeled without including the aorta in the computational domain (26, 37, 50). Adaptations in the inlet boundary condition may be necessary though (49).

We have not proven that our reduction method produces the optimal result. Other methods for lumping 1D model segments may lead to more complex outflow 0D models (e.g., those described in Refs. 19, 24, 32, 43) that yield smaller relative errors in pressure and the flow. In future work, we will extend our reduction methodology to account for inertial effects (e.g., using inductance terms) and more sophisticated tube laws [e.g., as proposed by Valdez Jasso et al. (44)], which may be more appropriate for hypertensive models (6).

It may not be possible to have a patient-specific model with all input parameters calculated from clinical measurements, but we need tools like the one presented here to eliminate unnecessary parameters and advance the usage of patient-specific simulations of arterial pressure and flow waveforms.

Given the excellent agreement between 1D and 3D pressure and flow waves in the aorta, as reported by Xiao et al. (52) under normal conditions, we believe that our methodology could be adapted to also determine the size of the computational domain in 3D blood flow modeling.

### Concluding Remarks

We have presented a new method for lumping peripheral 1D model vessels into 0D windkessel models that enables us to analyze the effect of network topology on pulse waveforms and determine the optimal number of arterial segments (and hence input parameters) required to simulate efficiently blood pressure and flow waveforms at the arterial sites that are relevant for a particular problem. This is important to simplify the computational domain while maintaining the precision of the numerical predictions.

## Appendix

We describe our novel methodology to lump a single nonlinear 1D model vessel of length *l* coupled with a linear three-element windkessel model at the outlet (Fig. 2*A*) into a linear two-element windkessel model (Fig. 2*C*). The vessel has a pressure and flow *p*_in_(*t*) and *q*_in_(*t*) at the inlet and *p*_out_(*t*) and *q*_out_(*t*) at the outlet. The outlet forms the inlet boundary conditions of the windkessel model, which has an outlet flow qout^(*t*) and constant pressure pout^.

The methodology requires linearization of *Eqs. 1* and *2* and transformation into 0D equations. In previous derivations of the linear 0D formulation from the nonlinear 1D equations, it was assumed constant cross-sectional area and material properties along the length of the arterial segment or an average cross-sectional area (2, 28, 31). In the following analysis we will not make such an assumption and will allow area and material properties to vary with the position along the vessel.

We consider the following linearized version of the 1D equations (4),

(11A) $$\frac{A_{\text{d}}}{\rho c_{d}^{2}}\frac{\partial p}{\partial t}+\frac{\partial q}{\partial x}=0,$$

(11B) $$\frac{\partial q}{\partial t}+\frac{A_{\text{d}}}{\rho }\frac{\partial p}{\partial x}=\frac{-2\left(\xi +2\right)\pi \mu q}{\rho A_{\text{d}}},$$

where we have used *Q* = *UA* and linearized about the diastolic conditions (*A*, *P*, *Q*) = (*A*_d_ + *a*, *P*_d_ + *p*, *q*), where *a*(*x*, *t*), *p*(*x*, *t*), and *q*(*x*, *t*) are the perturbation variables for area, pressure and flow rate. The physiological conditions in the arterial system are only weakly nonlinear; thus many characteristics can be captured by the linearized system (14). To continue we introduce the following theorem.

### Theorem 1

Mean Value Theorem (MVT): suppose *f*: [*b*_1_, *b*_2_] → ℜ is continuous and suppose *g* ≥ 0 is bounded and continuous on [*b*_1_, *b*_2_], then there is ζ ∈[*b*_1_, *b*_2_] such that

(12) $$\int_{b_{1}}^{b_{2}}f\left(x\right)g\left(x\right)\text{d}x=f\left(\zeta \right)\int_{b_{1}}^{b_{2}}g\left(x\right)\text{d}x,$$

and

(13) $$f\left(\zeta \right)=\frac{\int_{b_{1}}^{b_{2}}f\left(x\right)g\left(x\right)\text{d}x}{\int_{b_{1}}^{b_{2}}g\left(x\right)\text{d}x}.$$

Integrating the linearized 1D *Eqs. 11A* and *11B* along the *x*-axis yields

(14A) $$\int_{0}^{l}\;\left(\underset{a}{\underset{︸}{\frac{A_{\text{d}}}{\rho c_{\text{d}}^{2}}\frac{\partial p}{\partial t}}}+\underset{b}{\underset{︸}{\frac{\partial q}{\partial x}}}\right)dx=0,$$

(14B) $$\int_{0}^{l}\;\left(\underset{c}{\underset{︸}{\frac{1}{A_{\text{d}}}\frac{\partial q}{\partial t}}}+\underset{d}{\underset{︸}{\frac{1\partial p}{\rho \partial x}}}\right)dx=-\int_{0}^{l}\underset{e}{\underset{︸}{\frac{2\left(\xi +2\right)\pi \mu q}{\rho A_{d}^{2}}}}dx,$$

where we have divided the last equation by *A*_d_(*x*). We first evaluate both terms of Eq. 14*A*:

$$\left(a\right)\int_{0}^{l}\frac{A_{\text{d}}}{\rho c_{\text{d}}^{2}}\frac{\partial p}{\partial t}dx=\frac{1}{\rho }\frac{dp\left(\zeta_{1},t\right)}{dt}\int_{0}^{l}\frac{A_{\text{d}}}{c_{\text{d}}^{2}}dx;$$

$$\left(b\right)\int_{0}^{l}\frac{\partial q}{\partial x}dx=q_{\text{out}}-q_{\text{in}},\text{with}\;q_{\text{out}}=q(l,t)\;\text{and}\;q_{\text{in}}=q\left(0,t\right).$$

Since *p*(*x*, *t*) is continuous and $\frac{A_{\text{d}}}{c_{\text{d}}^{2}}$ is bounded and continuous, with *A*_d_(*x*) > 0 and *c*_d_(*x*) > 0 under physiological conditions, we can apply the MVT to (*a*), with ζ_1_∈[0, *l*].

We now evaluate each term of *Eq. 14B*:

$$\left(c\right)\int_{0}^{l}\frac{1}{A_{\text{d}}}\frac{\partial q}{\partial t}dx=\frac{dq\left(\zeta_{2},t\right)}{dt}\int_{0}^{l}\frac{1}{A_{\text{d}}}dx;$$

$$\left(d\right)\int_{0}^{l}\frac{1}{\rho }\frac{\partial p}{\partial x}dx=\frac{p_{\text{out}}-p_{\text{in}}}{\rho },\text{with}\;p_{\text{out}}=p(l,t)\;\text{and}\;p_{\text{in}}=p\left(0,t\right);$$

$$\left(e\right)-\frac{2\left(\xi +2\right)\pi \mu }{\rho }\int_{0}^{l}\frac{q}{A_{\text{d}}^{2}}dx=-\frac{2\left(\xi +2\right)\pi \mu }{\rho }q\left(\zeta_{3},t\right)\int_{0}^{l}\frac{1}{A_{\text{d}}^{2}}dx.$$

Since *q*(*x, t*) and $\frac{\partial q\left(x,t\right)}{\partial t}$ are continuous and $\frac{1}{A_{\text{d}}}$ and $\frac{1}{A_{\text{d}}^{2}}$ are bounded and continuous with *A*_d_(*x*) > 0 under physiological conditions, we can apply the MVT to (*c*) and (*e*) with ζ_2_ and ζ_3_ ∈[0, *l*] (28).

### Remark 1

Pulse waves propagate rapidly within the cardiovascular system (with velocities of about 4–15 m/s), while arterial lengths can vary between few millimeters up to ∼10 cm. As a result, pulse wave transit times within a vessel are small compared with the duration of the cardiac cycle. Thus, at any given time the space averaged values will be close to the pointwise ones and *p*(ζ_1_, *t*) = *p*_in_(*t*), *q*(ζ_2_, *t*) = *q*_out_ and *q*(ζ_3_, *t*) = *q*_out_ are reasonable (28).

*Equations 14A* and 14*B* become

(15A) $$\underset{a}{\underset{︸}{\frac{K_{1}}{\rho }\frac{dp_{\text{in}}}{\text{dt}}}}+\underset{b}{\underset{︸}{q_{\text{out}}-q_{\text{in}}}}=0,$$

(15B) $$\underset{c}{\underset{︸}{K_{2}\frac{dq_{\text{out}}}{\text{dt}}}}+\underset{d}{\underset{︸}{\frac{p_{\text{out}}-p_{\text{in}}}{\rho }}}=-\underset{e}{\underset{︸}{\frac{2\left(\xi +2\right)\pi \mu }{\rho }K_{3}q_{out}}}\;,$$

with

(16) $$K_{1}=\int_{0}^{l}\frac{A_{\text{d}}}{c_{\text{d}}^{2}}dx,\;\;\;K_{2}=\int_{0}^{l}\frac{1}{A_{\text{d}}}dx,\;\;\;K_{3}=\int_{0}^{l}\frac{1}{A_{\text{d}}^{2}}dx.$$

The term *C*_v_ = $\frac{K_{1}}{\rho }$ is the integrated arterial compliance of the 1D model vessel (Fig. 2*B*). We assume that the fluid inertia term *K*_2_$\frac{dq_{\text{out}}}{dt}$ (*Eq. 15B*) is negligible, since peripheral inertia has a minor effect on flow waveforms (2). Thus *Eqs. 15A* and *15B* become

(17A) $$C_{\text{v}}\frac{dp_{\text{in}}}{dt}+q_{\text{out}}-q_{\text{in}}=0,$$

(17B) $$p_{\text{out}}-p_{\text{in}}=-R_{\text{v}}q_{\text{out}},$$

with

(18) $$R_{\text{v}}=2\left(\xi +2\right)\pi \mu K_{3},$$

where *R*_v_ is the integrated resistance of the 1D model vessel (Fig. 2*B*).

We have shown that a 1D arterial segment can be reduced to a two-element windkessel model with resistance *R*_v_ and compliance *C*_v_. To close the problem we must also consider the following equation for the three-element windkessel model prescribed at the boundary (Fig. 2*A*),

(19) (1+R1R2)qout+CR1dqoutdt=Cdpoutdt+pout−pout^R2,

where output values of pressure and flow from the windkessel model are denoted as $\hat{p_{\text{out}}}$ and $\hat{q_{\text{out}}}$(*t*), respectively. By substituting *q*_out_(*t*) from *Eq. 17A* and *p*_out_(*t*) from *Eqs. 17B* into *Eq. 19* we obtain

(20) (1+R1R2+RvR2)qin−pin−pout^R2−(Cv(1+R1R2)+C+RvCvR2)dpindt+d(qin−Cvdpindt)dt(CR1+CRv)=0.

We make the assumption that the last term in *Eq. 20* is of negligible size compared with the other terms. This is deemed reasonable since

(21) $$\frac{d\left(q_{\text{in}}-C_{\text{v}}\frac{dp_{\text{in}}}{dt}\right)}{dt}=\frac{dq_{\text{out}}}{dt},$$

according to *Eq. 17A*, and consistent with our previous assumption of the inertia term *K*_2_$\frac{dq_{\text{out}}}{dt}$ being negligible. Each arterial segment acts as a buffer, due to its compliance; hence the rate of change of flow at the outlet $\frac{dq_{\text{out}}}{dt}$ is small compared with the other terms in *Eq. 20*.

Rearranging *Eq. 20* we get

(22) qin=pin−pout^R2+R1+Rv+(CvR2+CvR1+CR2+RvCv)(R2+R1+Rv)dpindt.

By comparing *Eq. 22* to the two-element windkessel equation (17, 48) we obtain

(23) qin=pin−pout^Rnew+Cnewdpindt,

where $\hat{p_{\text{out}}}$ is the value of pressure at which the outflow stops (most often approximated by the mean venous pressure), and *Eqs. 6A* and *6B* for *R*_new_ and *C*_new_. These denote the resistance and compliance of the two-element windkessel (Fig. 2*C*) that describes blood flow in the original 1D model vessel coupled to a three-element windkessel (Fig. 2*A*).

## GRANTS

We gratefully acknowledge the support of the Centre of Excellence in Medical Engineering [funded by the Wellcome Trust and Engineering and Physical Sciences Research Council (EPSRC) under Grant No. WT 088641/Z/09/Z] and the National Institute for Health Research (NIHR) Biomedical Research Centre at Guys and St Thomas' NHS Foundation Trust in partnership with King's College London. M. Willemet and J. Alastruey also received support from EPSRC Project Grant EP/K031546/1. J. Alastruey also acknowledges the support of a British Heart Foundation Intermediate Basic Science Research Fellowship (FS/09/030/27812).

## DISCLOSURES

No conflicts of interest, financial or otherwise, are declared by the author(s).

## AUTHOR CONTRIBUTIONS

Author contributions: S.E. and J.A. conception and design of research; S.E. performed experiments; S.E. analyzed data; S.E., M.W., P.C., and J.A. interpreted results of experiments; S.E. and M.W. prepared figures; S.E. and J.A. drafted manuscript; S.E., M.W., P.C., and J.A. edited and revised manuscript; S.E., M.W., P.C., and J.A. approved final version of manuscript.
